# Donor, Not Recipient, Liver Immune Status Index Determines Early Recurrence in Living Donor Liver Transplantation

**DOI:** 10.31662/jmaj.2024-0037

**Published:** 2024-04-01

**Authors:** Etsuro Hatano

**Affiliations:** 1 Department of Surgery, Graduate School of Medicine, Kyoto University, Kyoto, Japan

**Keywords:** Hepatocellular carcinoma, Liver transplantation, Biomarker, Early recurrence

Liver transplantation is the most curative treatment for hepatocellular carcinoma (HCC) accompanied by liver cirrhosis. According to guidelines in Western countries, local therapy such as liver resection for HCC associated with liver cirrhosis is uncommon. Liver transplantation, which can also cure underlying liver disease that causes HCC, is the mainstream radical treatment. However, in advanced HCC, recurrence after liver transplantation―that is, the presence of circulating tumor cells (CTCs) and extrahepatic micrometastasis at the time of liver transplantation is a problem. The conventional approach is to create the Milan criteria, 5-5-500 criteria, and HALT-HCC score based on tumor diameter, tumor number, and tumor markers to clarify the tumor image that will not recur and prevent recurrence. Previous research has predominantly attributed the recurrence after liver transplantation in HCC to tumor characteristics.

Imaoka et al. demonstrated the importance of the donor liver immune status index (LISI), not the recipient LISI, in influencing patient outcomes in living donor liver transplantation (LDLT), particularly in the context of advanced HCC ^(1)^. LISI, consists of BMI, serum albumin levels, and fibrosis-4 (FIB-4) index. The FIB-4 index was calculated using age, aspartate aminotransferase, platelet count, and alanine aminotransferase. The LISI score, which is an accurate predictor of TRAIL expression, specifically in liver natural killer (NK) cells with interindividual variability, represents a valuable parameter for stratifying high-risk patients with vascular invasion following hepatic resection ^(2)^. The authors have proposed the significant role of liver NK cells in maintaining intrahepatic immunity.

In this study ^(1)^, after propensity score matching, the recurrence free survival (RFS) rate was significantly worse in the high-donor LISI group than in the low donor LISI group. The high-donor LISI group showed a higher frequency of early recurrences approximately 1 year after transplantation, particularly in cases of advanced HCC. The role of donor LISI has become increasingly important, particularly considering the known function of NK cells in targeting CTCs.

When considering the indications for liver transplantation for advanced HCC, if the donor’s LISI is high, the risk of recurrence will be high, and the indication for transplantation should be abandoned; if there is a living donor with a low LISI, LDLT should be considered. There may be an advantage in being able to make offers. This groundbreaking report shows that the donor’s antitumor capacity of liver NK cells influences the recurrence of transplanted recipients. However, is it possible to exclude high-LISI donors from donor candidates during the donor selection process? The high-donor LISI defined in this study accounted for 38% of the total. Conversely, in countries with many brain-dead donors, donor options might be available.

This study has several limitations. This single-center study examining experiences with LDLT is based on a relatively small patient cohort. Approximately 37% of the patient cohort received adoptive immunotherapy, a treatment for which safety has been established in phase I clinical trials. However, its efficacy in enhancing therapeutic outcomes, especially in the context of LDLT for HCC, has yet to be fully determined. Therefore, a validation study is required.

Donor LISI plays a significant role in increasing the risk of early recurrence after LDLT for HCC. Such a tailored approach to donor selection and adoptive immunotherapy could be useful for expanding the criteria for liver transplantation for HCC.

## Article Information

### Conflicts of Interest

None

### ORCiD iD

Etsuro Hatano: https://orcid.org/0000-0003-3407-1918
